# Supplementary material for: Exploring Protein-Inorganic Hybrid Nanoflowers and Immune Magnetic Nanobeads to Detect Salmonella Typhimurium
Source: Nanomaterials (Basel). 2018 Dec 4;8(12):1006. doi: 10.3390/nano8121006 (PMC6316584; doi:10.3390/nano8121006)
Supplement: Supplementary file 1 [file nanomaterials-08-01006-s001.pdf]

Exploring Protein-Inorganic Hybrid Nanoflowers and Immune Magnetic Nanobeads to Detect *Salmonella* Typhimurium

Lei Wang[1], Xiaoting Huo[1], Ruya Guo[2], Qiang Zhang[3], Jianhan Lin[1,*]

[1]Key Laboratory of Agricultural Information Acquisition Technology, Ministry of Agriculture, China Agricultural University, Beijing 100083, China; wanglei123@cau.edu.cn (L.W.); huoxiaoting@cau.edu.cn (X.H.); jianhan@cau.edu.cn (J. L.)

[2]Key Laboratory of Modern Precision Agriculture System Integration Research, Ministry of Education, China Agricultural University, Beijing 100083, China; guoya@cau.edu.cn (R. G.)

[3]Department of Biosystems Engineering, University of Manitoba, Winnipeg, MB R3T 2N2, Canada; Qiang.Zhang@umanitoba.ca (Q. Z.)

*Correspondence: jianhan@cau.edu.cn; Tel.: +86-10-6273-7599

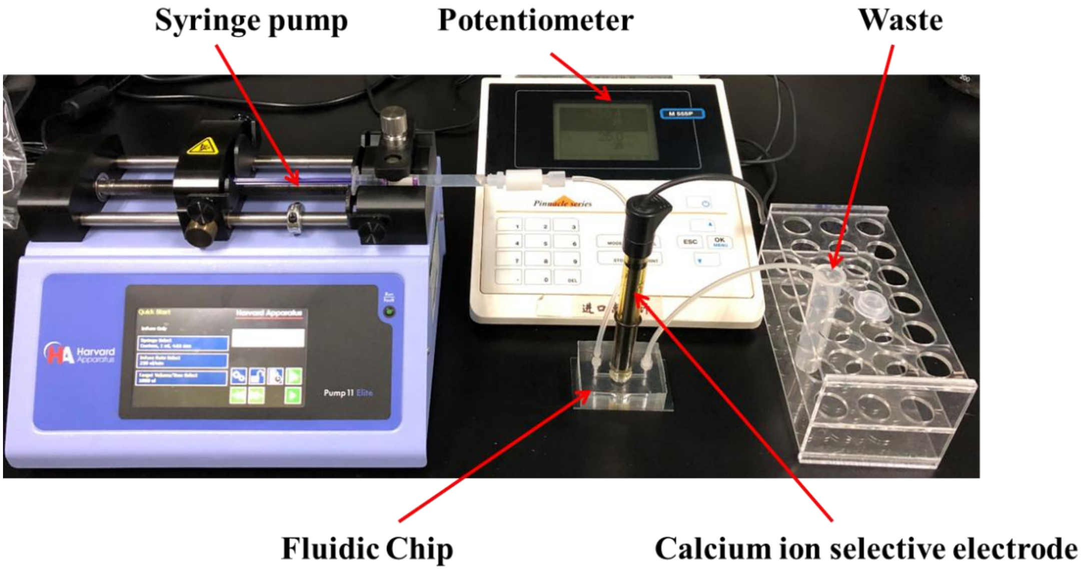

(a) The platform for proof of the proposed assay

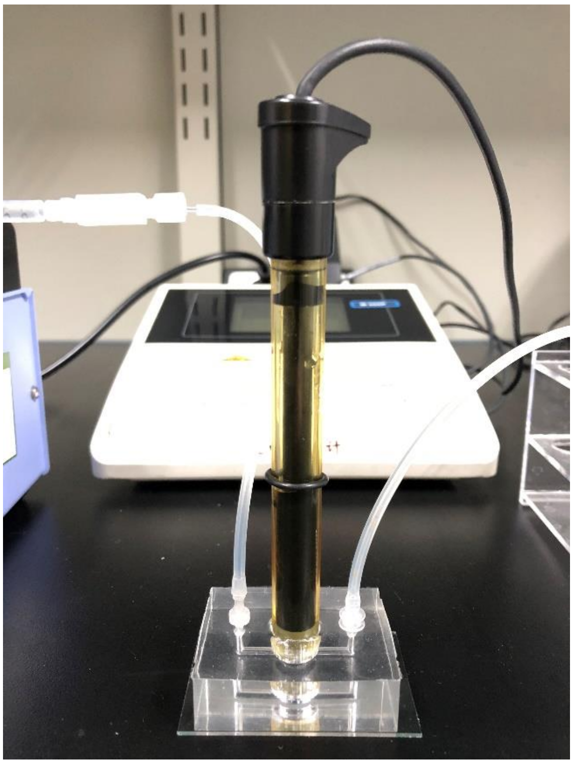

(b) The fluidic chip with the Ca-ISE

Fig. S1

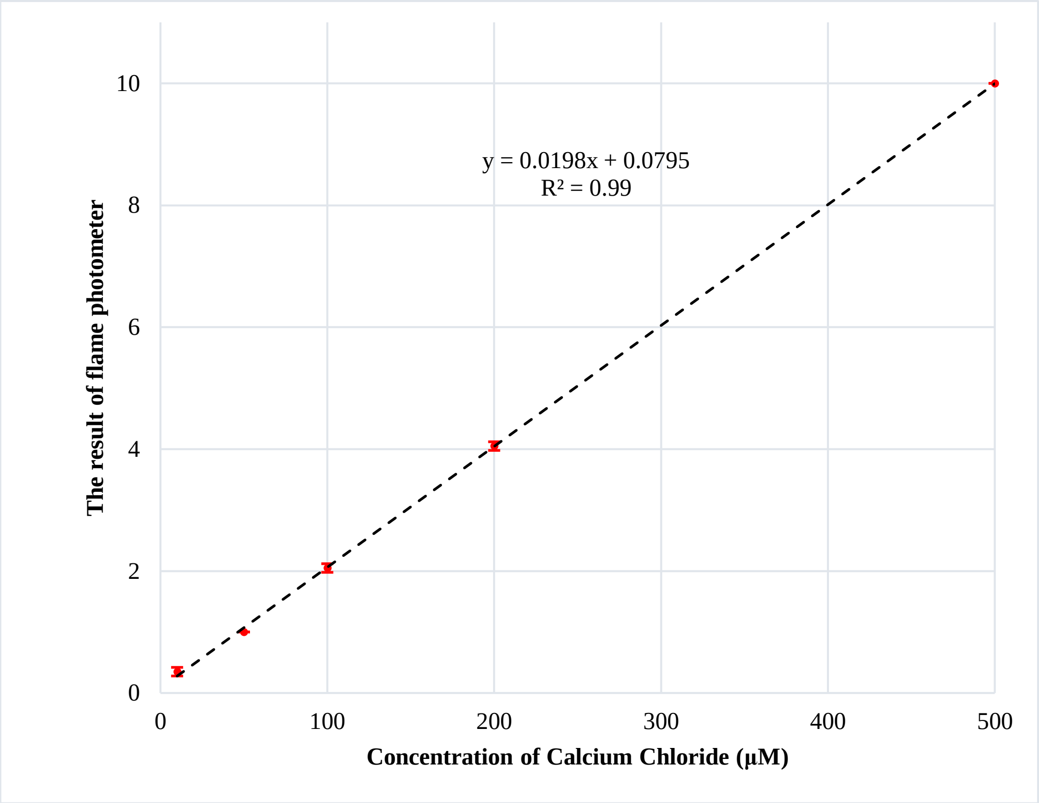

Fig. S2 Calibration curve of the flame photometer (N=3).
